# A new species of *Pupinidius* Möllendorff, 1901 (Gastropoda, Stylommatophora, Enidae) from Jiuzhaigou, Sichuan, China

**DOI:** 10.3897/BDJ.12.e123920

**Published:** 2024-07-16

**Authors:** Zhong-Guang Chen, Yu-Ting Dai, Xiao-Ping Wu, Jiao Jiang, Shan Ouyang

**Affiliations:** 1 School of Life Sciences, Nanchang University, Nanchang, China School of Life Sciences, Nanchang University Nanchang China; 2 Zhejiang Museum of Natural History, Hangzhou, China Zhejiang Museum of Natural History Hangzhou China

**Keywords:** conchology, taxonomy, land snail, Sichuan

## Abstract

**Background:**

The genus *Pupinidius* Möllendorff, 1901 is endemic in China and Nepal and consists of 15 species. China is the distribution centre of it with 12 species being recorded.

**New information:**

A new species, *Pupinidiuspulchellus* Chen, Dai, Wu & Ouyang sp. nov. is described from Jiuzhaigou, Sichuan, China. It can be distinguished from congeneric species by the shell with wide and distinct radial stripes; the thin, slightly reflexed and reddish-brown peristome; the unpointed apex; the unfused A-1 and A-2; the sub-globular and well defined bursa copulatrix; the unexpanded diverticle and the presence of epiphallic caecum.

## Introduction

The genus *Pupinidius* Möllendorff, 1901 of family Enidae Woodward, 1903 is a group of medium-sized land snails characterised by the short cylindrical shell (Wu and Zheng 2009, Wu 2018). It is widely distributed from the eastern to southern edges of the Qinghai Tibet Plateau (Kuznetsov and Schileyko 1997, Kuznetsov and Schileyko 1999, Wu and Zheng 2009, Wu 2018). Currently, it consists of 15 species, 12 of which are recorded in China: *P.anocamptus* (Möllendorff, 1901), *P.chrysalis* (Annandale, 1923), *P.gregorii* (Möllendorff, 1901), *P.latilabrum* (Annandale, 1923), *P.melinostoma* (Möllendorff, 1901), *P.nanpingensis* (Möllendorff, 1901), *P.obrutschewi* (Sturany, 1900), *P.porrectus* (Möllendorff, 1901), *P.wenxian* Wu & Zheng, 2009, *P.pupinella* (Möllendorff, 1901), *P.pupinidius* (Möllendorff, 1901 and *P.streptaxis* (Möllendorff, 1901) (Kuznetsov and Schileyko 1997, Kuznetsov and Schileyko 1999, Wu and Zheng 2009, Wu 2018). These species are distributed in limited areas within several dry-hot river valleys located at the eastern edge of the Qinghai Tibet Plateau including the Bailongjiang River Valley, the Daduhe River Valley, the Jinshajiang River Valley and the Lancangjiang River Valley (Wu and Zheng 2009, Wu 2018). The valleys are separated by mountains with elevations exceeding 5000 m and *Pupinidius* is limited to the lower part of the valley and does not extend to the alpine meadows in the upper part. The highly fragmented distribution area nurtures the high species diversity. Amongst the valleys, the Bailongjiang River Valley has the highest diversity of *Pupinidius* species, with a total of nine species recorded.

The section of the Bailongjiang River (known as Baishuijiang River in Sichuan) Valley between Jiuzhaigou County and Longnan City is renowned for its exceptionally diverse enid land snails. Since the late 19^th^ and early 20^th^ centuries, several explorers, missionaries and naturalists have collected and described numerous species of family Enidae in the area (Gredler 1898, Sturany 1899, Möllendorff 1901, Yen 1939, Wu and Zheng 2009, Wu 2018). The Jiuzhaigou County is located at the most upstream part of Baishuijiang River and has many unique enid species. However, only one species of the genus *Pupinidius*, including two subspecies, has been recorded here (Wu and Zheng 2009, Wu 2018). The species diversity of *Pupinidius* in the region is much lower than in other sections of the Bailongjiang River Valley, which may suggest the presence of other species that have yet to be discovered. In this study, we described a new species of *Pupinidius* from Jiuzhaigou County, based on shell morphology and genitalia anatomy. The discovery fills the gap between the distribution areas of *Pupinidius* and enhances our understanding of its diversity.

## Materials and methods

Specimens were collected from Sichuan of China in 2021–2023. Living specimens were initially frozen at -20℃ for 12 hours and subsequently thawed at room temperature for 12 hours to facilitate the extraction of soft parts. The soft parts were then fixed in 70% ethanol. Empty shells were cleaned, dried and preserved at 4℃. Measurements were taken with digital callipers to the nearest 0.1 mm. Whorls were counted as described by Kerney and Cameron (1979). Terminology follows Wu (2018) who defined the genital atrium as proximal. Photographs were taken by camera (A6500, Sony, Minato City, Japan).

Abbreviations: NCU_XPWU: Laboratory of Xiao-Ping Wu, Nanchang University (Nanchang, Jiangxi, China); At: atrium; AR: retractor muscle of the appendicular branch; A-1: most proximal section of penial appendix; A-2: penial appendix section between and thicker than A-1 and A-3, usually bulb-shaped; A-3: section of the penial appendix connecting proximally A-2 and distally A-4; A-4: thinnest part of the penial appendix between A-5 and A-3; A-5: distal part of the penial appendix, more or less swollen; BC—bursa copulatrix; BCD: bursa copulatrix duct; D: diverticle of the bursa copulatrix duct; Ep: epiphallus; EpC: epiphallic caecum; Fl: flagellum; FO: free oviduct; P: penis; PC: penial caecum; PR: retractor muscle of the penial branch; Va: vagina; VD: vas deferens.

## Taxon treatments

### Pupinidius pulchellus

Chen, Dai, Wu & Ouyang sp. nov.

15DC97A0-724B-5BDE-B5D4-C34D381E9D05

B467E5EA-915C-4135-B83C-4829E243ADAA

#### Materials

- **Type status:** Holotype. **Occurrence:** occurrenceID: ABC12FFA-2635-557D-AB5F-6A083198829E; **Location:** locationID: country: China; stateProvince: Sichuan; verbatimLocality: Jiuzhaigou County, Shuanghe Town [双河镇], Ancient Cliff Roadway [古栈道]; verbatimLatitude: 33°9'57"N; verbatimLongitude: 104°15'53"E; **Identification:** identifiedBy: Zhong-Guang Chen & Yan-Shu Guo; **Event:** eventDate: July, 2021; **Record Level:** institutionID: NCU; collectionID: 24XPWAN201
- **Type status:** Paratype. **Occurrence:** occurrenceID: 7D24CBE3-B57F-552E-9A92-092E9E266A1F; **Location:** locationID: country: China; stateProvince: Sichuan; verbatimLocality: Jiuzhaigou County, Shuanghe Town [双河镇], Ancient Cliff Roadway [古栈道]; verbatimLatitude: 33°9'57"N; verbatimLongitude: 104°15'53"E; **Identification:** identifiedBy: Zhong-Guang Chen & Yan-Shu Guo; **Event:** eventDate: July, 2021; **Record Level:** institutionID: NCU; collectionID: 24XPWAN202–220
- **Type status:** Paratype. **Occurrence:** occurrenceID: A68B5445-93C1-50A3-97FE-B40AAA5972A6; **Location:** locationID: country: China; stateProvince: Sichuan; verbatimLocality: Jiuzhaigou County, Shuanghe Town [双河镇], Ancient Cliff Roadway [古栈道]; verbatimLatitude: 33°9'57"N; verbatimLongitude: 104°15'53"E; **Identification:** identifiedBy: Zhong-Guang Chen, Meng-Hua Li & Jin-Sheng Mou; **Event:** eventDate: August, 2023; **Record Level:** institutionID: NCU; collectionID: 24XPWAN221–250
- **Type status:** Paratype. **Occurrence:** occurrenceID: FAE18991-9CE2-55AE-9287-3B1F1CF2852B; **Location:** locationID: country: China; stateProvince: Sichuan; verbatimLocality: Jiuzhaigou County, Chaimenguan [柴门关]; verbatimLatitude: 33°5'4"N; verbatimLongitude: 104°22'10"E; **Identification:** identifiedBy: Zhong-Guang Chen & Yan-Shu Guo; **Event:** eventDate: July, 2021; **Record Level:** institutionID: NCU; collectionID: 24XPWAN251–260

#### Description

Shell (Fig. 1a). Shell cylindrical-conical, with apex abruptly pointed; shell most swollen (broadest) at body whorl, dextral, medium, thick, solid, semi-transparent, glossy, speckled, not spirally grooved; 5.75–6.25 whorls. Whorls rather flattened, not shouldered. Protoconch smooth, polished. Post-nuclear whorls smooth. Growth lines distinct. Suture white, normal, without narrow band beneath it. Body whorl gradually ascending towards aperture, rounded at periphery. Aperture in a plane, truncate-ovate, oblique, without tooth, with weak angular tubercle, completely adnate to body whorl. Peristome not connected; reddish-brown, slightly thickened, expanded, slightly reflexed. Parietal callus distinct. Columellar margin reflexed. Umbilicus opened, slit-shaped. Shell multicoloured, post-nuclear whorls with reddish-brown background and irregular, wide yellowish-white stripes; apex region reddish-brown. Height 20.7–24.6 mm, width 10.4–12.2 mm.

**Figure 2.**
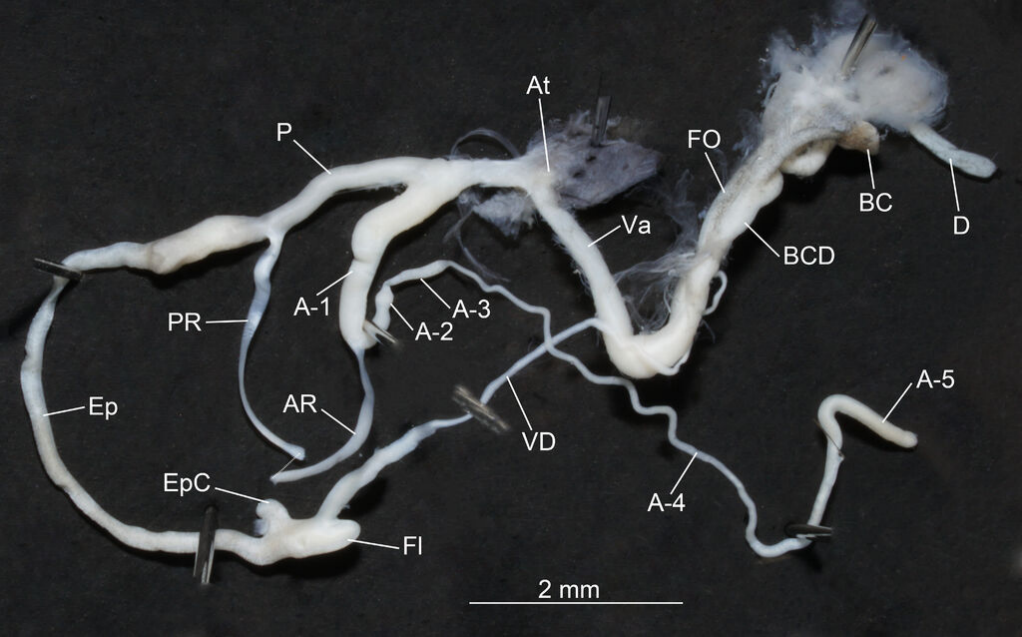
Genitalia anatomy of *Pupinidiuspulchellus* sp. nov.

Genitalia (Fig. 2). Vas deferens short, swollen proximally; entering epiphallus apically with distinct demarcation. Epiphallus long; cylindrical and of uniform thickness; straight; externally smooth. Epiphallic caecum present; blunt apically; located near vas deferens entrance. Flagellum short; conical; proximally expanded; with tip pointed. Penis with terminal entrance of epiphallus; clavate; distally tapering. Penial caecum absent. Penial appendix long; branched off from penis at some distance from atrium; divided into sections including A-1, A-2, A-3 and A-4+A-5. A-1, A-2 and A-3 not fused. Boundary between A-4 and A-5 indistinct. A-5 long; convoluted. Penial retractor long; biramous; attaching to penis distally and to A-1 of penial appendix; with penial retractor arms arising from diaphragm closed to each other. Additional retractor other than penial or appendicular absent. Muscular band connecting vagina and epiphallus absent. Atrium short; without retractor. Free oviduct shorter than vagina. Vagina long; swollen; straight; unpigmented. Bursa copulatrix duct short; proximally straight. Bursa copulatrix sub-globular, with stalk; without apical ligament; normal in size; with long neck; well defined. Diverticle normally present; longer than reservoir; unexpanded. Bursa copulatrix and diverticle distinguishable; forked more distally from their base.

#### Diagnosis

Shell with wide and distinct radial stripes; peristome thin, slightly reflexed and reddish-brown; A-1 not fused with A-2; bursa copulatrix sub-globular and well defined; diverticle unexpanded; epiphallic caecum present.

#### Etymology

The specific name is made from the Latin *pulchellus* for pretty, alluding to the pretty stripes on the shell of this species. Vernacular name: 丽纹蛹巢螺 (Pinyin: li wen yong chao luo).

#### Distribution

**Figure 3.**
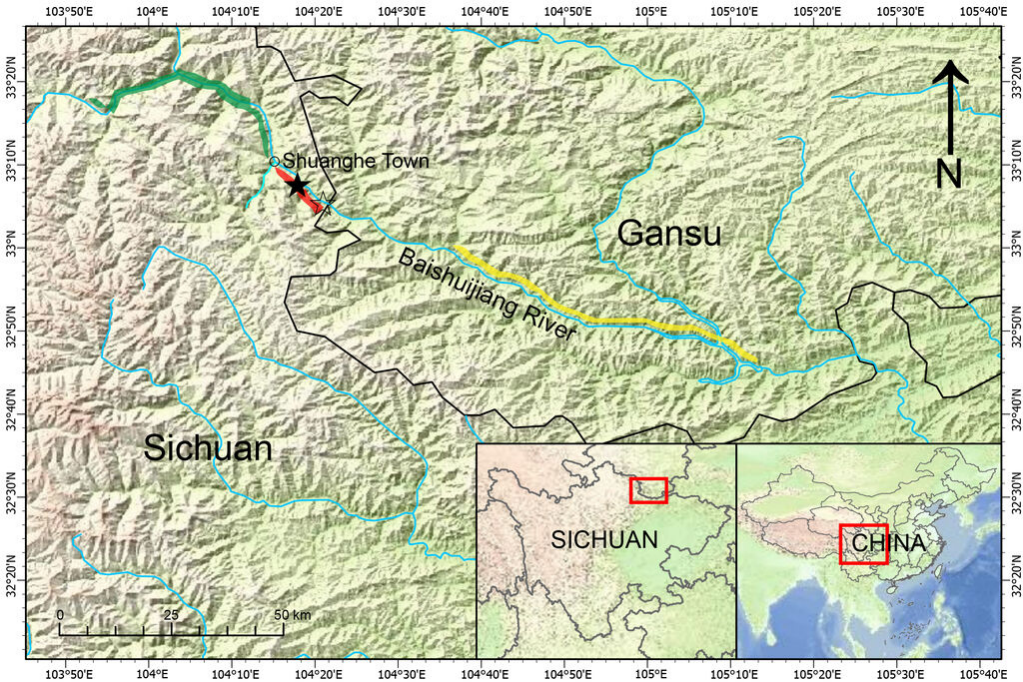
Distribution of *Pupinidiuspulchellus* sp. nov. and its neighbouring congeners. Red shadow shows *Pupinidiuspulchellus* sp. nov., green shadow shows *Pupinidiusnanpingensis*, yellow shadow shows *Pupinidiusmelinostoma*, solid star shows the Ancient Cliff Roadway, hollow star shows the Chaimenguan.

**Figure 4.**
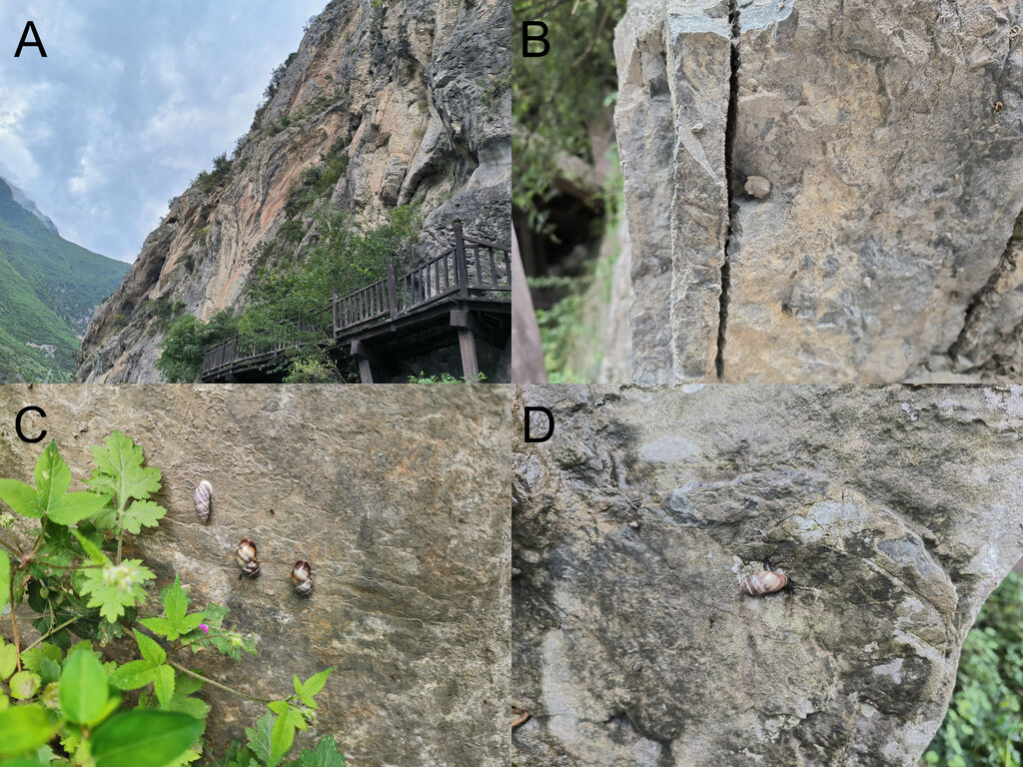
Environment of the type locaity and living specimens. **A** Ancient Cliff Roadway; **B** juvenile; **C** dormant adults; **D** crawling adult.

*Pupinidiuspulchellus* sp. nov. is only distributed within a few kilometres from the Ancient Cliff Roadway to Chaimenguan on the right bank of Baishuijiang River in Jiuzhaigou (Figs 3, 4). *Pupinidiusnanpingensis* is distributed at the west of its habitat, while *P.melinostoma* is found at the east. The distributions of three species do not overlap and are separated by the Baishuijiang River and its tributary. The boundary between the distribution ranges of the new species and *P.nanpingensis* is a small unnamed tributary with a deep valley that joins the Baishuijiang River at Shuanghe Town, with the new species downstream, while the *P.nanpingensis* is upstream. The boundary between the distribution ranges of the new species and *P.melinostoma* is the Baishuijiang River, with the new species on the right bank, while the *P.melinostoma* is on the left bank.

#### Ecology

*Pupinidiuspulchellus* sp. nov. inhabits on steep rock cliff together with *Pseudonapaeusberesowskii* (Möllendorff, 1902), *Laeocathaicadistinguenda* Möllendorff, 1899 and *Serina* sp. and almost never descending to the ground. Juveniles wrap themselves up by mud (Fig. 4).

#### Taxon discussion

The new species can be easily distinguished from *P.pupinella*, *P.anocamptus*, *P.chrysalis*, *P.latilabrum*, *P.nanpingensis*, *P.wenxian*, *P.gregorii*, *P.porrectus*, *P.himalayanus*, *P.siniayevi* and *P.tukuchensis* by the shell with wide and distinct radial stripes (vs. with extremely wide radial stripes nearly covering the entire post nuclear whorls in *P.pupinella*; without or almost without stripes in *P.anocamptus*, *P.chrysalis*, *P.latilabrum*, *P.nanpingensis*, *P.wenxian*, *P.himalayanus*, *P.siniayevi* and *P.tukuchensis*; with spiral stripes in *P.gregorii* and *P.porrectus*). It is similar with *P.melinostoma*, *P.obrutschewi*, *P.pupinidius* and *P.streptaxis* by the similar radial striped shell, but differs from them by the thinner and reddish-brown peristome (vs. thicker and white) and the not fused A-1 and A-2 (vs. fused). It is further distinguished from *P.melinostoma* by the thicker stripes on shell, the sub-globular and well-defined bursa copulatrix (vs. tubular and not well defined), the unexpanded diverticle (vs. expanded) and the presence of epiphallic caecum (vs. absence); from *P.obrutschewi* by the unpointed apex (vs. pointed); from *P.pupinidius* by the slightly reflexed peristome (vs. strongly reflexed); from *P.streptaxis* by the taller shell.

## Supplementary Material

XML Treatment for Pupinidius pulchellus

## Figures and Tables

**Figure 1a.**
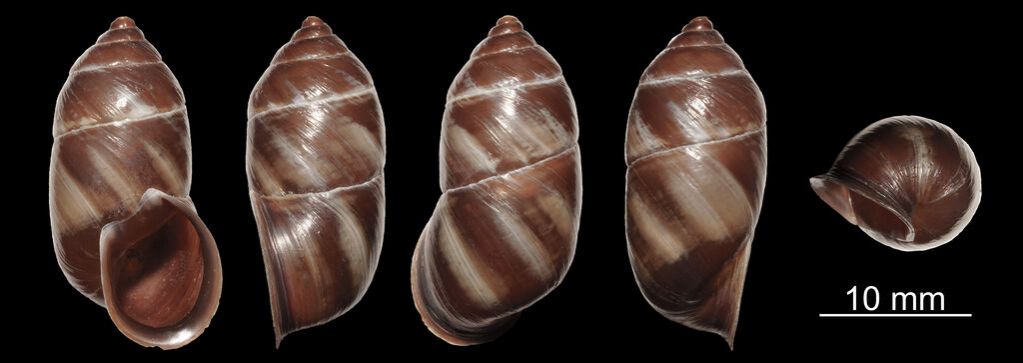
*Pupinidiuspulchellus* sp. nov., holotype;

**Figure 1b.**
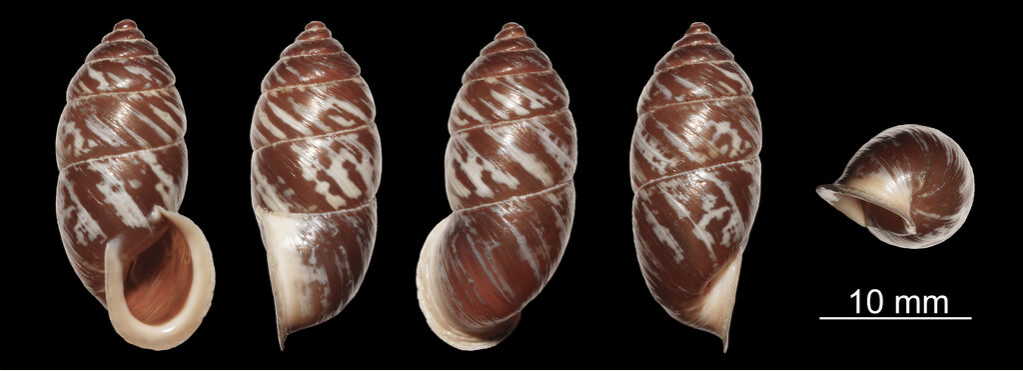
*Pupinidiusmelinostoma*.
